# The Role of Influenza in the Delay between Low Temperature and Ischemic Heart Disease: Evidence from Simulation and Mortality Data from Japan

**DOI:** 10.3390/ijerph13050454

**Published:** 2016-04-28

**Authors:** Chisato Imai, Adrian G. Barnett, Masahiro Hashizume, Yasushi Honda

**Affiliations:** 1 School of Public Health and Social Work, Queensland University of Technology, 60 Musk Avenue, Brisbane QLD 4064, Australia; a.barnett@qut.edu.au; 2 Department of Pediatric Infectious Diseases, Institute of Tropical Medicine, Nagasaki University, 1-12-4 Sakamoto, Nagasaki 852-8523, Japan; hashizum@nagasaki-u.ac.jp; 3 Faculty of Health and Sport Science, The University of Tsukuba, Comprehensive Research Building D, 1-1-1 Tennoudai, Tsukuba 305-8577, Japan; honda@taiiku.tsukuba.ac.jp

**Keywords:** cold, temperature, ischemic heart disease, influenza, mortality

## Abstract

Many studies have found that cardiovascular deaths mostly occur within a few days of exposure to heat, whereas cold-related deaths can occur up to 30 days after exposure. We investigated whether influenza infection could explain the delayed cold effects on ischemic heart diseases (IHD) as they can trigger IHD. We hypothesized two pathways between cold exposure and IHD: a direct pathway and an indirect pathway through influenza infection. We created a multi-state model of the pathways and simulated incidence data to examine the observed delayed patterns in cases. We conducted cross-correlation and time series analysis with Japanese daily pneumonia and influenza (P&I) mortality data to help validate our model. Simulations showed the IHD incidence through the direct pathway occurred mostly within 10 days, while IHD through influenza infection peaked at 4–6 days, followed by delayed incidences of up to 20–30 days. In the mortality data from Japan, P&I lagged IHD in cross-correlations. Time series analysis showed strong delayed cold effects in the older population. There was also a strong delay on intense days of influenza which was more noticeable in the older population. Influenza can therefore be a plausible explanation for the delayed association between cold exposure and cardiovascular mortality.

## 1. Introduction

Both extreme high and low temperatures increase the risk of cardiovascular diseases (CVD), but there is a marked difference in how quickly the events occur. The impacts of extreme heat occur quickly (1–2 days), whereas the impacts of cold usually remain for up to a month [1,2]. Though many studies have reported these distinct differences, the cause of the difference has not been investigated in detail. 

For cold exposure, mounting observational evidence points to influenza as a trigger of ischemic heart disease (IHD), as demonstrated by the overlapping seasonal patterns of influenza and IHD [3,4,5]. The biological mechanisms that potentially explain the link between those diseases is the contribution of acute respiratory infections to blood coagulation and inflammation in the vasculature [6]. Supported by physiological reasons, a study reported a risk reduction in IHD after influenza vaccination in a high risk population [7]. However, the idea has raised questions because the highest CVD mortality occurs within a few days of cold exposure, but this is not long enough for respiratory infections to develop [8]. Considering the time between infections to manifestations of IHD, it points towards one possible mechanism whereby influenza infections play a role in the delayed development of IHD after cold exposure, which could potentially explain the prominent difference from the rapid heat effects on IHD. 

**Figure 1:**
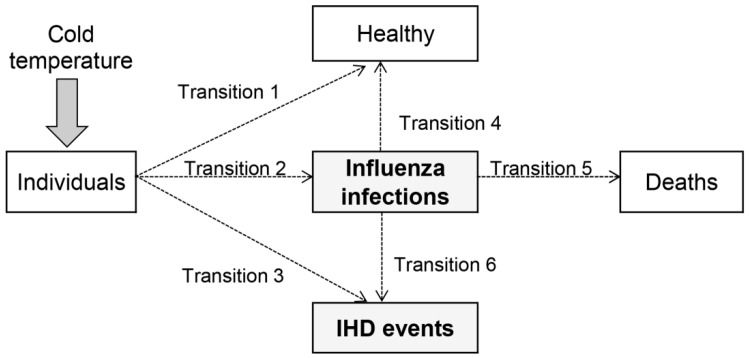
A multi-state competing risk model for influenza infections and IHD mortality or morbidity incidence. Individuals enter the model after being exposed to cold temperatures. First, individuals follow one of transition pathways 1 to 3. Those who followed Transition 2 and entered the state of influenza infections take one of Transitions 4 to 6. The primary IHD occurs when individuals take Transition 3 directly after cold exposure, whereas the secondary IHD occurs if individuals go through Transitions 2 and 6.

In the present study, we attempted to explore the plausibility of the hypothesis that influenza infection could act as an intermediate state that causes the delayed cold effects on IHD. Specifically, the aim of this study is to investigate the hypothesis that there are two routes between cold exposure and IHD: a direct pathway and an indirect pathway via influenza infection (Figure 1). We refer to these two routes as primary and secondary IHD, respectively. Since there are few studies done in this context, the present study was conducted primarily for hypothesis building with theoretical and empirical evaluations. For comparison we also examined the effect of extreme heat.

## 2. Materials and Methods 

The analyses primarily consisted of two components. The first component was a theoretical evaluation using simulation, and the second was an empirical evaluation using the actual data from Japan. Each result and consistency in findings between those analyses were examined whether they support our hypothesis. In following section, we first address the methods of simulation in which competing risks and multi-state models are briefly introduced in the beginning. Then we describe the details of the data and analysis methods used for empirical assessments.

### 2.1. Simulation Using Multi-State Models with Competing Risks

#### 2.1.1. Competing Risks and Multi-State Models

Competing risks occur where multiple and mutually exclusive events occur from one starting state (*i.e.*, condition) [9]. Often, one type of event is singled out as the event of interest, however more than one type of events usually play a role in the process of the event development. Those other event types that may prevent the event of interest from occurring are called competing risks. Competing risks analysis estimates the probability of the event of interest in the presence of competing risks. 

Multi-state models are a type of survival analysis that can incorporate competing risks. They are useful for time to event data in which individuals start from one initial state and eventually end up in one or more states. In between, intermediate states can exist and be revisited possibly more than once [10,11,12]. Multi-state models are particularly useful when one is interested in the event that happens after the non-fatal event (*i.e.*, intermediate state). 

Altogether, multi-state models of competing risks have the advantage of reflecting the reality where individuals are often subjected to more than one event, providing biological insights to the processes of disease development and recovery over time. The methods also allow estimates of median time to events and event probabilities [10]. 

In the present study, we used this method to examine if influenza is an intermediate state that creates a delayed cold effect on IHD, especially by estimating the time for IHD events via direct and indirect pathways through influenza infection between cold and IHD. Our multi-state model is shown in Figure 1. We assume influenza infection and IHD are competing risks as they both can be impacted by cold exposure but mutually exclusive events. Because not all individuals develop the diseases, an unaffected state (*i.e.*, remain healthy) was also considered in the transitions after exposed to cold (Transition 1 to 3 respectively). After influenza infection, one was expected to follow one of the transitions; recovery to healthy, unrecovered (death), or development of IHD (Transition 4 to 5). The primary IHD is when the disease development takes the direct route via Transition 3, and the secondary IHD occurs through the indirect route via Transitions 2 and 6. 

#### 2.1.2. Simulation for IHD Morbidity

Simulation adds to our understanding of how competing risks could proceed. In our study, we simulated each event transition in Figure 1 with the aim of examining how the processes of the direct and indirect IHD events create the overall delayed effect of cold temperature. If our hypothesis or the structure of multi-state competing risks model is theoretically correct, simulation will yield the overall IHD with delays that are consistent with the actually exiting IHD data.

Event times were generated using the exponential distribution as the standard distribution for competing models with constant event probability [9]. Transition states were estimated using the multinomial distribution to randomly select competing states. The starting state was an extremely cold day. In order to simulate event times, the mean event times (*i.e.*, daily probability of events) were required and thus estimated from previous studies and reports. The mean event times for individuals to take either one of Transitions 1 to 3 in Figure 1 were estimated based on the health quality study [13] and the reports of outpatient statistics from the National Federation of Health Insurance Societies and the Ministry of Health, Labour and Welfare (MHLW) in Japan [14,15]. Based on those previous publications, the mean rate 0.55 stays healthy (Transition 1) as the rate of the population claims no health issues. For Transition 2 and 3, the daily probabilities of influenza infection and IHD morbidity was estimated 0.0104 and 0.000735 respectively based on the actual case reports. The transition from influenza infections to recovery (Transition 4) had a mean rate of 0.14 (1 event per 7 days) meaning that the average recovery time from influenza was a week. The times to death (Transition 5) and to development of IHD from influenza (Transition 6) were estimated based on previous studies [16,17]. To avoid heterogeneity, the studies for the probability estimates were, where possible, from the same region and time (*i.e.*, targeting February 2012 in Japan). However, due to limited studies, probability estimates for some transitions required using studies outside the target. In particular, because there are few studies in regard to the mortality rate from influenza infection to IHD (Transition 6), the estimate for the event transition alternatively used an IHD morbidity study. The details for all transitions are in Figure S1 in the Supplementary Material. 

For simplicity, we assumed no secondary transmissions of influenza, and that cases contracted influenza on the first day. We examined a range of assumptions concerning event times and transition rates. We summarized the simulated data using histograms of daily event numbers and cumulative risk curves over time. We then compared these plots and summary statistics of median times to the well-established pattern of the delayed IHD risk in order to assess our hypothesized model.

### 2.2. Mortality Data from Japan

#### 2.2.1. Data

Daily nationwide mortality in Japan from 1973 to 2012 for IHD and pneumonia and influenza (P&I) were obtained from the Japanese MHLW. IHD was classified as the International Classification of Diseases (ICD)-8 and -9 410–414, and ICD-10 I20–I25; pneumonia as ICD-8 and -9 480–486 and ICD-10 J12–J18; and influenza as ICD-8 470–474, ICD-9 487, and ICD-10 J10–J11. Though pneumonia can be caused by different pathogens, several studies have used P&I mortality as a reliable proxy for influenza activity [18,19,20,21,22]. Age groups of 15–64 years, and 65 or older were used. Temperature data were obtained from the Japan Meteorological Agency. The daily mean temperature averaged over the 47 prefectural capitals (for the two prefectures, the closest cities to the capitals) was used in this analysis. The statistical summaries for IHD and P&I mortality, and mean temperatures are available in the Supplementary Material (Tables S1 and S2). 

#### 2.2.2. Statistical Analysis

##### Cross-Correlations

Analyses for the data from Japan were conducted with cross-correlations and time series regression models. First, cross-correlation explored whether the data follows the time-ordering of events in Figure 1 that hypothesize the intermediate role of influenza infection. A cross-correlation analysis is a simple but useful method to identify lagged relationships between two time series. We used the method to identify the associations and time lags among IHD, P&I, and temperature. The correlations were estimated for warm (April to September) and cold (October to following March) seasons to look for differences in the delayed association by season. Data after the year 2009 were excluded in the cross-correlations due to pandemics of a novel influenza virus (H1N1) which created unusual seasonality.

##### Time Series Analysis 

For in-depth exploration, we investigated the role of influenza infection in associations between IHD and temperature with time series regression analysis. The analysis examined how the delayed response of IHD mortality to temperature would be changed by considering P&I mortality. The main aim of this analysis was to provide empirical estimates of the delayed effects of temperature that we could then compare with our simulations from the theoretical model (Figure 1). This analysis used a generalized linear model with a quasi-Poisson distribution allowing for overdispersion, and distributed lag nonlinear models to examine the delayed and non-linear association (DLNM) [23]. The model is:

(1) $$\begin{array}{cc} & Y_{t\;}\sim Poisson\left(\mu_{t},\;\theta_{t}\right)\\ \log\left(\mu_{t}\right)=DOW_{t} & +ICD_{t}+cb(TMP_{t-l})+ns\left(time,\;df\right)\\ & +intx\left(TMP_{t-l},intense\;flu\;day_{t}\right) \end{array}$$

where $Y_{t}$ is the daily number of IHD deaths on day t, θ is an overdispersion parameter, DOW is a categorical variable for day of the week. A categorical variable ICD is to account for the transition periods from ICD-8 to ICD-10. The periods for the disease classification system were taken into consideration as the base of disease occurrence could alter due to the different diagnosis coding (Figure S2  in the Supplementary Material). TMP is mean daily temperature and l is the lag days. cb(TMP) is a cross-basis function for daily temperature parameterized with natural cubic spline terms on temperature and the delayed response [24]. Following previous studies in which associations and delayed effects between IHD mortality and temperature were U-, V-, or J-shaped [25,26], the smooth function used three equally placed knots for temperature and lag. The lag duration was from 0 to 30 days. ns(time, df) is a smooth function of time using natural cubic splines to control for seasonality and long-term trends in deaths [27]. For this smoothing function on time, three degrees of freedom (df) per year was used after conducting sensitivity analyses to select the optimum degrees of freedom based on Akaike’s Information Criterion (see Figure S3  in the Supplementary Material). 

To investigate if the period of the highest influenza season increased the average delay between low temperatures and IHD mortality, we used the interaction term (intx) between the cross-basis function of temperature and a dichotomous variable of intense days of influenza (intense days are 1, otherwise 0). The intense days were defined as days when the number of P&I deaths exceeded the 80th percentile of the distribution in each epidemic year which starts in October. Since this cut-off point does not perfectly separate days with and without active influenza transmission, sensitivity analyses were conducted using cut-offs of the 70th percentile and 90th percentile, but these alternative cut-offs did not significantly change the results.

The current model (Equation (1)) does not include a variable for intense influenza days because it was not statistically significant and our focus was not the direct impact of influenza but its combined role with IHD. 

The temperature–flu interaction was tested with an *F*-test. To visually show the impact of the interaction we plotted the delayed association between temperature and IHD deaths during intense and non-intense days. We expected that during intense influenza days the average delayed association with temperature would be longer as there would be more transitions on the delayed pathway (Figure 1). All statistical analyses were conducted using the R software version 3.1.1 (R Development Core Team, Vienna, Austria) [28] with the “DLNM” package to fit the distributed lag non-linear model [24]. 

## 3. Results

### 3.1. Simulation

**Figure 2:**
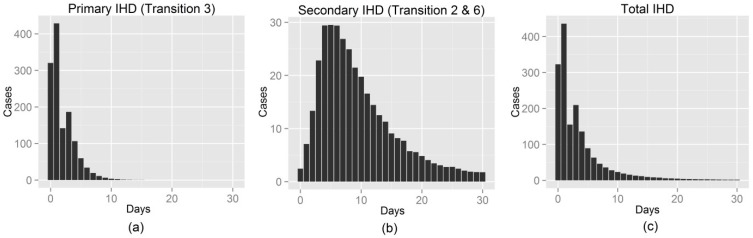
Simulated distributions of time to IHD incidence via: (**a**) the direct pathway (Transition 3); (**b**) the indirect pathway (Transitions 2 + 6); (**c**) Results averaged over 100 simulations of cohorts of 100,000 people.

The simulated distributions of times to primary and secondary (*i.e.*, by influenza infections) IHD are in Figure 2. The primary IHD observations take place quickly and the majority of cases occur within 10 days, whereas IHD manifested through influenza infections peak at 4–6 days followed by delayed cases up to 20–30 days. Though the number of secondary IHD incidence was smaller than that of primary IHD, the secondary pathway added exponential delays to the total IHD. 

**Figure 3:**
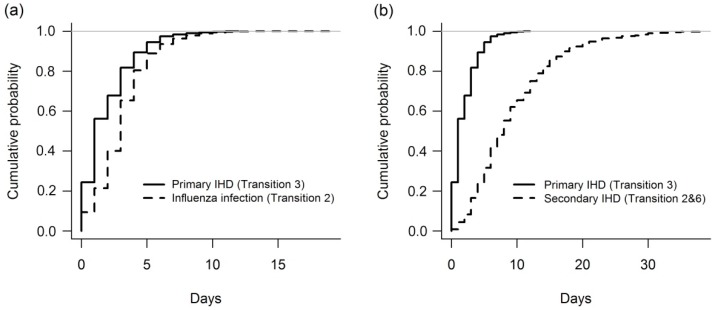
Cumulative probability plots of IHD incidence and influenza infections. Cumulative probability plots of (**a**) primary IHD incidence and influenza infections; and (**b**) primary and secondary IHD incidence. Results shown for a single randomly selected simulation.

The cumulative probability plots (Figure 3) further illustrate the immediate and delayed times of the two pathways between cold exposure and IHD events. Most secondary IHD occurs by 20 days, but a few cases linger up to 30 days.

### 3.2. Japan’s Mortality Data

#### 3.2.1. Cross-Correlations

**Figure 4:**
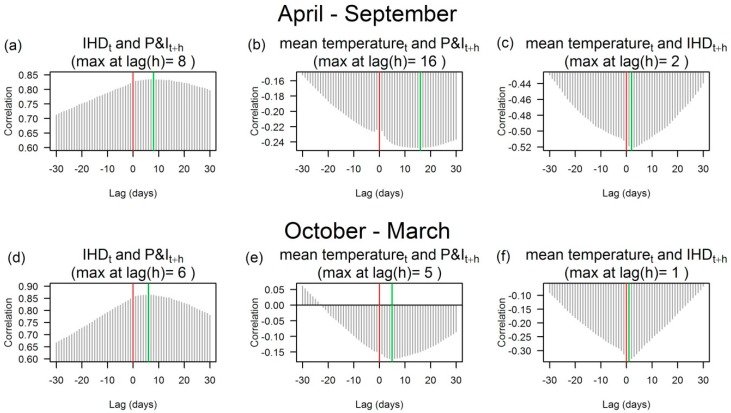
Cross-correlations of daily P&I and IHD deaths and daily temperatures among the population aged 65 or older in Japan, 1973–2009. Cross-correlations identify the lags (*h*) of one variable ($x_{t+h}$) relative to the other ($y_{t}$). The red vertical line at lag 0 is for the same day (*t*). The green vertical line highlights the lag h with the largest cross-correlation. The correlograms show the cross-correlations of (**a**) IHD and P&I deaths; (**b**) mean temperature and P&I deaths; (**c**) mean temperature and IHD deaths in a warm season (April to September); (**d**) P&I deaths; (**e**) mean temperature and P&I deaths; and (**f**) mean temperature and IHD deaths in a cold season (October to March).

We assessed cross-correlations up to 30 day lags by age group. The characteristics of the results were very similar between young (aged 15–64) and aged (65 or older) populations. The results of aged population are shown in Figure 4. See Figure S4  in the Supplementary Material for ages 15–64.

P&I mortality and mean temperature were inversely correlated, because P&I mortality increases in winter. P&I mortality lagged mean temperature, but the lag time of the highest correlation was shorter in the cold season (5 day lag) than the warm season (16 day lag). The lag response for high IHD mortality occurring after low temperatures was relatively quick (1 or 2 days) in both seasons. 

For associations between IHD and P&I mortalities, the correlations were strong positive (*r* ≥ 0.65) throughout the period as expected from their remarkably similar seasonal patterns. As the lag response to mean temperature was shorter with IHD morality than P&I mortality, the highest cross-correlations showed P&I mortality lagged IHD mortality by 6 and 8 days in the cold and warm seasons, respectively. This pattern of P&I lagging IHD supports our hypothesis of secondary IHD via the intermediate state of influenza infection can take longer than primary IHD after cold exposure, and thus contributes to delayed cold effects on IHD. 

#### 3.2.2. Time Series Analysis

**Figure 5:**
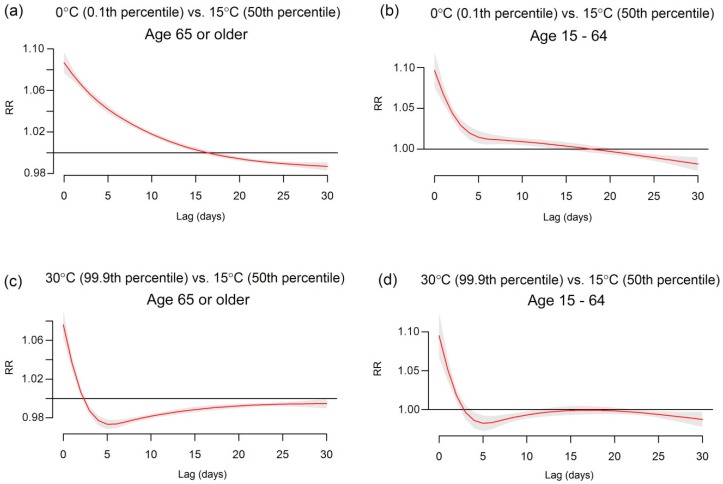
The estimated delayed effects of extreme temperatures on IHD deaths for population aged 65 years or older and age of 15–64 in Japan, 1973–2012. The red lines show the mean association and the grey areas are 95% confidence intervals. The plots show the cold effects on (**a**) aged 65 years or older; (**b**) age of 15–64; and the heat effects on (**c**) aged 65 years or older; (**d**) age of 15–64.

Associations between IHD mortality and temperature were first assessed without influenza (*i.e.*, no interaction term in Equation (1)). The relative risks (RRs) for extreme low and high temperatures (0 and 30 °C) are in Figure 5. These temperatures are approximately the 0.1 and 99.9 percentile of temperature. Both extreme temperatures are associated with an immediate increase in risk (approximately up to 5 days) for those aged 15–64 years, whereas for those aged 65 or older only high temperatures show a similar immediate increase. For the older population, the extreme cold effect had an attenuated risk over time with delayed impacts until approximately 16 days. There was some mortality displacement after the initial rise in risk among the older population in April to September. We would not expect a delayed association during the season as influenza infection is not active in summer, meaning very few cases travel the secondary route via influenza (Figure 1).

The empirical patterns in risk shown in Figure 5 are similar to our theoretical patterns in Figure 2. Our theoretical model of risk decreases gradually to day 30, as does the risk in the elderly. Our theoretical model has a “bump” in risk at day 3 due to the rise of deaths from the delayed transition. This peak also appeared in the empirical data when the degrees of freedom for temperature and lag were increased to allow a more flexible association (see Figure S5  in the Supplementary Material). For the younger population the empirical risk of cold is relatively short-lived with the majority occurring within 10 days (Figure 5). This is similar to our theoretical model with only an immediate effect of cold exposure (Figure 1), suggesting that the lack of a long delayed effect in the younger population could be because IHD deaths due to influenza infections are not an important factor. In order to show the similarity between theoretical and empirical results, the lag response curves for their estimated risks of cold temperature are provided in Figure S6  in the Supplementary Material. 

**Figure 6:**
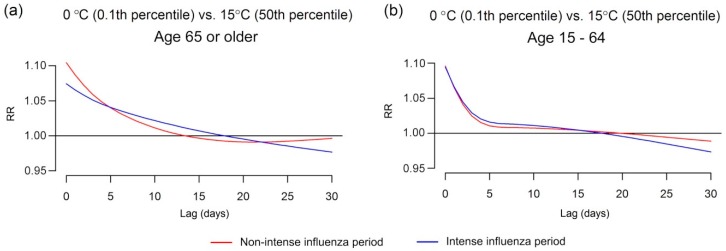
The extreme cold effect on IHD deaths on intense and non-intense days among (**a**) age 65 or older and (**b**) age 15–64.

We added an interaction between temperature and influenza epidemics in order to see whether this changed the lagged association (Figure 6). The cold effect during intense days of influenza was more delayed than during non-intense days among the population 65 or older. For the population aged 15–64 years there was little visual difference in risk by intense and non-intense days, which is further evidence of the unimportance of influenza infections in the younger population. The *F*-test to examine the interactions were statistically significant in both populations, however, the interaction in the older population (*F* = 22.00, *p* < 0.001) explained more variance than the younger population (*F* = 3.64, *p* < 0.001). Comparing these results to our theoretical model (Figure 1), the delay in risk becomes longer when there are more active influenza transmissions and more transitions to the influenza infections (Transition 2), although this is not the case for the younger population possibly because more of them recover (Transition 4).

## 4. Discussion

In the simulation results, most primary IHD cases occurred within 10 days whereas the majority of secondary IHD cases took approximately 20 days (Figure 2). The total time for all IHD cases to occur was a predominantly exponential decline with delays up to 20–30 days, as similarly observed in many observational studies [29,30,31]. The interaction analysis in time series models showed that the effect of extreme cold temperature on IHD deaths during intense influenza days was more delayed than non-intense days. 

Furthermore, the interaction analysis showed that the risk during non-intense influenza days fell below the risk of intense days after 5 days among the older population. It illustrated the shifts of the dominant factor for IHD from cold exposure to influenza infections after the time lag. Interestingly, the time lag also coincided with the approximate maximum time of the occurrence of the majority IHD cases in the younger population in which influenza did not significantly change the delayed cold effect. These results are consistent with cold being primarily accountable for the primary IHD in both age groups, and for the older population, influenza infection creates delayed IHD deaths. 

Age as an important modifier is not surprising, considering the vulnerability of aging population due to underlying age-related risks of cardiovascular disease (e.g., hypertension, diabetes, high level of serum total cholesterol) [32]. Coagulating agents such as fibrinogen also increased by respiratory infections cause more chances of arterial thrombogenesis for the elderly than for younger adults [33]. The time lag that influenza infection rises as the predominant factor for IHD deaths is also worth noting. In previous observational studies the role of influenza as a confounder of the association between cardiovascular disease and temperature has not been completely accepted and remains ambiguous [34,35]. Our result indicated that the impact of influenza might be overshadowed if the optimal lag timings are not appropriately chosen in time series analyses. 

Though these results were exactly what we would expect based on our hypothesis with influenza as the key intermediate state causing the delays, delayed cold effects on IHD were still observed even after taking into account influenza infection. One possible reason is that cold in itself may have delayed impacts. There is incomplete knowledge as to how delayed IHD cases are biologically explained via cold exposure, but cumulative effects of cold in the development of IHD has been reported [36]. The other explanation for the delay is unmeasured intermediating effects. Though we focused on influenza in the present study, other respiratory tract and bacterial infections could also trigger a systematic inflammatory response that increases the risk of IHD [37]. For a more complete understanding of the mechanism of the delayed cold effects it would be useful for future studies to account for these possible factors, but this would require detailed individual data on deaths, including secondary causes and lengths of stay in hospital prior to death.

There are limitations we need to acknowledge. First, our analysis was conducted with P&I mortality as a proxy of influenza activity. The actual number of the infected incidence among IHD cases would have provided more accurate pictures of the impacts of influenza. However, getting such data is difficult since 30%–50% of influenza cases are non-febrile [38,39,40]. In addition, as far as P&I mortality captures the daily variations of influenza among the population in which IHD cases occur, it is still sufficient to assess the impacts of influenza on IHD in our time series analysis. Secondly, no time lags of influenza incidences was assumed in the time series analysis although our hypothesis illustrated influenza infection is on the pathway from cold temperature exposure to secondary IHD events (Figure 1). Influenza infections on the previous days of IHD events are, however, highly correlated with the current day’s infections (e.g., correlation is consistently ≥0.94 with up to each previous 14 day), that is, the results are less likely to change even considering time lags of influenza incidences. 

Other important limitations include the underlying assumption for competing risks process that the risks of events and exposures remain unchanged over time [12], which is rarely true in reality. For instance, we did not consider secondary transmissions for influenza infections, though this would likely create more delayed cases. Our competing risks model was a simplified picture of reality where other events may also be involved, but the model captures the most significant events in the context of short-term cold effects. Acute respiratory infections and cardiovascular diseases are responsible for a large proportion of increasing morbidity and mortality in winter, and their short-term associations with cold exposure are widely recognized from epidemiological evidence and physiological plausibility [41,42,43]. There is a limitation in the published studies available which do not give the perfect information to inform our simulation or provide information on interactions with age. However, the simulated data provides the best current understanding in the likely course of the disease occurrence as it was consistent with empirical analysis findings and biologically compelling. We did not include other factors potentially associated with IHD, in particular air pollution [43,44]. However the role of air pollution as a confounder of temperature is dubious [45].

## 5. Conclusions 

The present study does not convey conclusive casual interpretations since the aim was to examine the hypothesis with simulation tests and empirical data. However, given the consistent results, our study highlights the potential role of influenza infections in the delayed cold effect due to secondary IHD. 

The role of cold temperature and influenza infection in IHD deserves further investigation, as increasing our understanding of the underlying cause of delays of IHD in winter can have large public health implications given the high number of winter deaths. Not only is IHD the world’s leading cause of deaths [46], but the aging population continues to increase globally from 11.7% in 2013 to 21.1% by 2050 [47]. This study provides some important public health implications: (1) Prevention efforts for cardiovascular diseases such as reducing age-related risk factors may prevent both primary and secondary IHD; (2) Prevention of influenza infection may reduce the total burden of cardiovascular disease; (3) These prevention measures need to be intensified in the elderly population. 

Cold temperatures are a much bigger killer than heat [42], and this may worsen if extremely cold winters become more frequent in some parts of the world [48]. Though heat impacts are often emphasized in the light of global warming, the future changes in patterns of weather and global population mean that researchers and governments need to continue to pay attention to the health risks of low temperatures.

## Supplementary Materials

The following are available online at www.mdpi.com/1660-4601/13/5/454/s1, Figure S1: A multi-state competing risk model for influenza infections and IHD mortality or morbidity incidence, Figure S2: Time series plots for IHD and P&I mortality, and mean temperature in Japan, 1973–2012, Figure S3: Sensitivity analysis for the degrees of freedom for the smoothing function on time and the extreme cold effect on IHD deaths on intense and non-intense days among age 65, Figure S4: Cross-correlations among population aged 15–64, Figure S5: Increased degrees of freedom for the splines for lag and temperature, Figure S6: Lag responses for estimated risks of a cold effect on IHD based on simulation and empirical data for (a) all age group and (b) age 65 or older, Table S1: Statistics of IHD and P&I mortality, Table S2: Statistics of daily mean temperature in Japan.

## Abbreviations

The following abbreviations are used in this manuscript:

ICD	International Classification of Diseases
IHD	Ischemic heart diseases
MHLW	Ministry of Health, Labour and Welfare
P&I	Pneumonia and influenza
RR	Relative risks
