# Supplementary material for: The Role of Influenza in the Delay between Low Temperature and Ischemic Heart Disease: Evidence from Simulation and Mortality Data from Japan
Source: Int J Environ Res Public Health. 2016 Apr 28;13(5):454. doi: 10.3390/ijerph13050454 (PMC4881079; doi:10.3390/ijerph13050454)
Supplement: Supplementary file 1 [file ijerph-13-00454-s001.pdf]

# Supplementary Materials: The Role of Influenza in the Delay between Low Temperature and Ischemic Heart Disease: Evidence from Simulation and Mortality Data from Japan

**Chisato Imai, Adrian G. Barnett, Masahiro Hashizume and Yasushi Honda**

## 1. Simulation for Competing Risks

Random observations (*N* = 1,000,000) were generated from an exponential distribution using a multinomial distribution for the first transitions (Transitions 1 to 3 in Figure S1 below). Then, observations assigned to influenza infections were again randomly sampled for the second phase of transitions (Transitions 4 to 6). This was repeated 100 times and averaged.

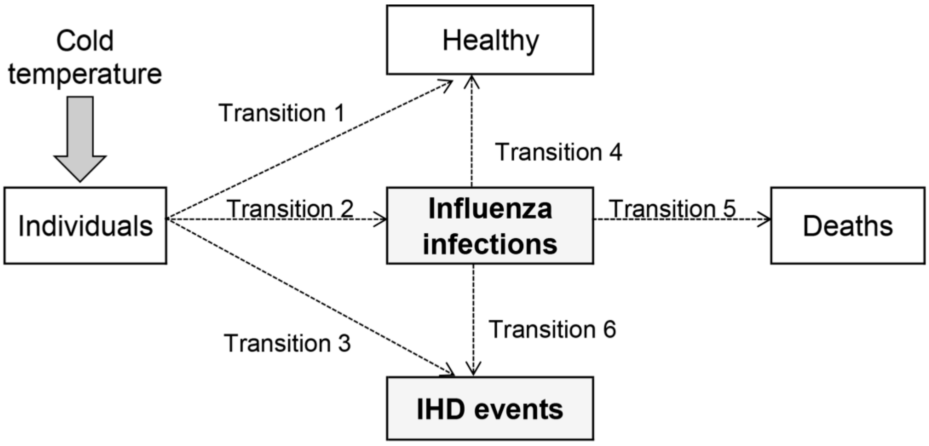


**Figure S1.** A multi-state competing risk model for influenza infections and ischemic heart diseases (IHD) mortality or morbidity incidence.

### *1.1. Estimations of Event Times*

We used previous studies to estimate the rate for the exponential distributions. The mean event time is the inverse of the rate.

#### 1.1.1. Transition 1: Individuals to Stay Healthy

The rate for the exponential distribution was estimated from a study for health-related quality life in Japan. In the study [1], a representative population was examined by the international standard survey called EuroQol (EQ-5D) to measure the different dimensions of health status (e.g., morbidity, pain/discomfort, and anxiety/depression) and 45.6% of the study population reported problems in any dimensions of health status. Therefore, we assume that one can maintain the condition with no health issues is 55% of daily probability.

#### 1.1.2. Transition 2: Individuals Infected with Influenza

The rate for the exponential distribution was estimated from the number of outpatient records diagnosed with influenza infections reported by National Federation of Health Insurance Societies [2]. During the epidemic season 2011–2012, February had the highest number of influenza diagnoses (approximately 410,000) among insurance holders (approximately 1,363,000). Therefore, the daily rate was estimated as 0.0104 (1.04%) ≈ 410,000 ÷ 1,363,000 ÷ 29 days. Since incubation period of influenza is on average 2 days [3], the first 2 days were assumed to be one-fifth of the mean rate.

#### 1.1.3. Transition 3: Individuals to Ischemic Heart Diseases (IHD)

The rate for the exponential distribution was estimated from the patient survey data from the Japanese Ministry of Health, Labor and Welfare [4]. The daily outpatient for ischemic heart diseases (IHD) was approximately 49 per 100,000 (0.00049% or 0.049%) in October, 2011. Given that our mortality data shows an approximate 50% increase of cases when compared with following February in 2012, the outpatient rate was also doubled (0.000735% or 0.0735%).

#### 1.1.4. Transition 4: Influenza Infections to Recovery (Healthy)

With an assumption that recovery takes one week, the rate for the exponential distribution was estimated as 0.14 (14%) ≈ (1 event ÷ 7 days).

#### 1.1.5. Transition 5: Influenza Infections to No Recovery

In Hong's study [5], four cases among 635 laboratory confirmed influenza cases were reported as deaths within an average of 8 days. Therefore, the mean rate for the exponential distribution was estimated as 0.00079(0.079%) ≈ (4 events ÷ 635 events) ÷ 8 days.

#### 1.1.6. Transition 6: Influenza Infections to IHD

Smeeth's study [6] reported that 332 of 20,921 cases with acute respiratory infections developed a myocardial infarction within 3 days. The rate for the exponential distribution was therefore estimated as 0.005(0.5%) ≈ (332 ÷ 3 days) ÷ 20,921.

## 2. Statistics for IHD and Pneumonia and Influenza (P&I) Mortality

Daily minimum (min), maximum (max) and mean mortality are calculated based on the data from 1995–2012 which corresponds the period of ICD-10 (Table S1). The total cases were based on the study period of 1973–2012.

**Table S1.** Statistics of IHD and P&I mortality in Japan.

| Age Stratification | Min | Max | Mean | Total |
|---|---|---|---|---|
| **All age** | | | | |
| IHD deaths | 111 | 389 | 200 | 2,394,012 |
| P&I deaths | 128 | 752 | 255 | 2,809,532 |
| **Age 14–65** | | | | |
| IHD deaths | 11 | 72 | 31 | 405,378 |
| P&I deaths | 1 | 57 | 11 | 164,460 |
| **Age 65 or older** | | | | |
| IHD deaths | 87 | 342 | 171 | 1,988,395 |
| P&I deaths | 115 | 702 | 259 | 2,618,718 |

## 3. Statistics of Daily Mean Temperature in Japan

**Table S2.** Statistics of daily mean temperature in Japan.

| Daily Mean | Min | Max | Mean | Standard Deviation |
|---|---|---|---|---|
| **Temperature (°C)** | −1.2 | 30.0 | 15.5 | 8.2 |

## 4. Time Series Plots for Mortality Data and Temperature

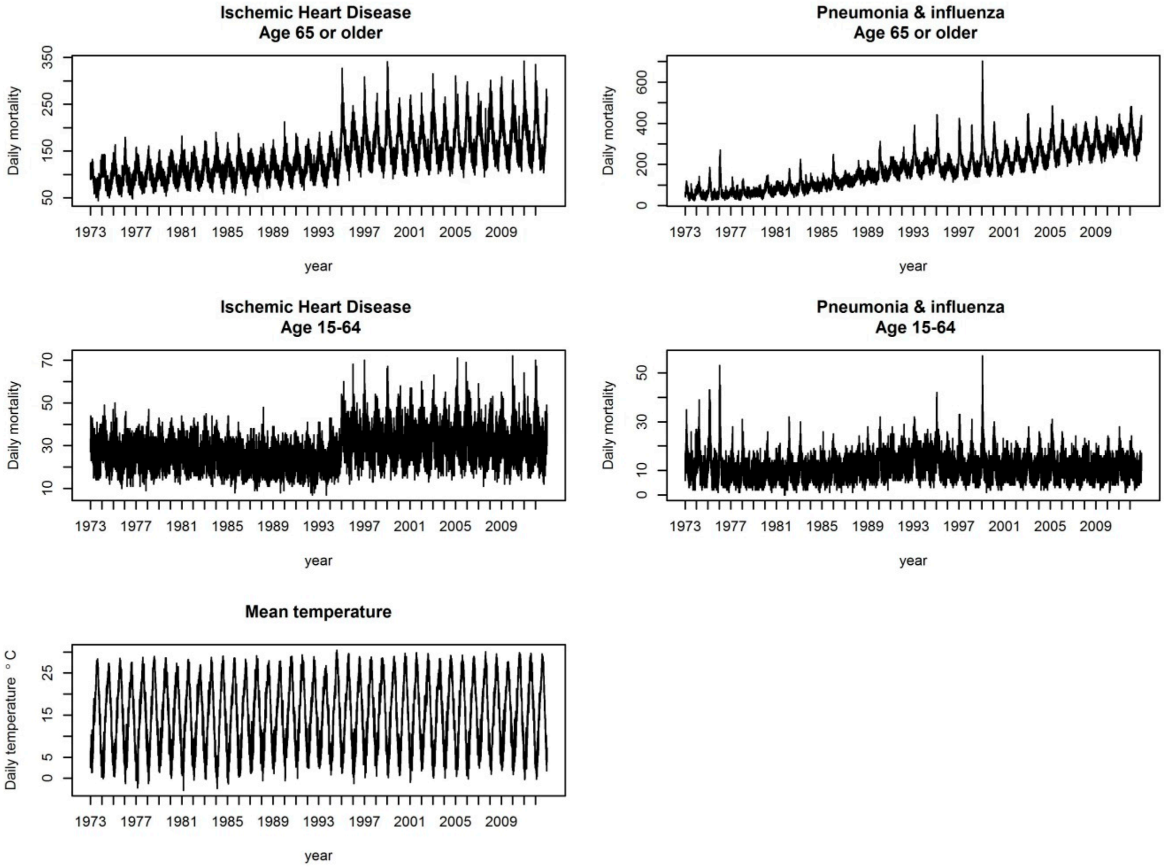


**Figure S2.** Time series plots for IHD and P&I mortality, and mean temperature in Japan, 1973–2012.

## 5. Sensitivity Analysis for the Degrees of Freedom for the Smoothing Function on Time

We examined whether changing the number of degrees of freedom per year could impact on our interaction results. The graphs in Figure S3 show that, regardless of different number of the degrees of freedom, the patterns of different lag responses between intense and non-intense influenza days were consistent, proving the robustness of our results.

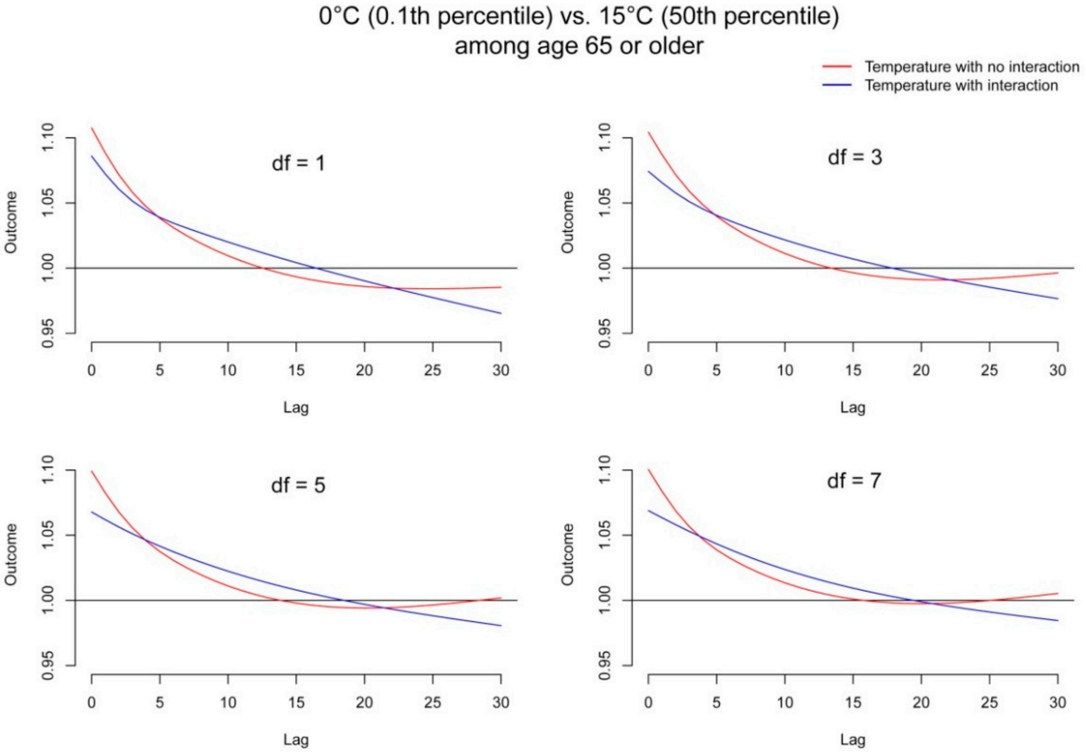


**Figure S3.** Sensitivity analysis for the degrees of freedom for the smoothing function on time and the extreme cold effect on IHD deaths on intense and non-intense days among age 65.

## 6. Cross-Correlations among Population Aged 15–64 Years

Figure S4 shows the cross-correlations in daily P&I and IHD deaths and daily temperatures among the population aged 15–64 in Japan, 1973–2009. Negative lags correspond to P&I deaths before IHD deaths, and positive lags correspond to P&I deaths after IHD deaths. The red vertical line at zero is for the same day. The green vertical line highlights the largest cross-correlation.

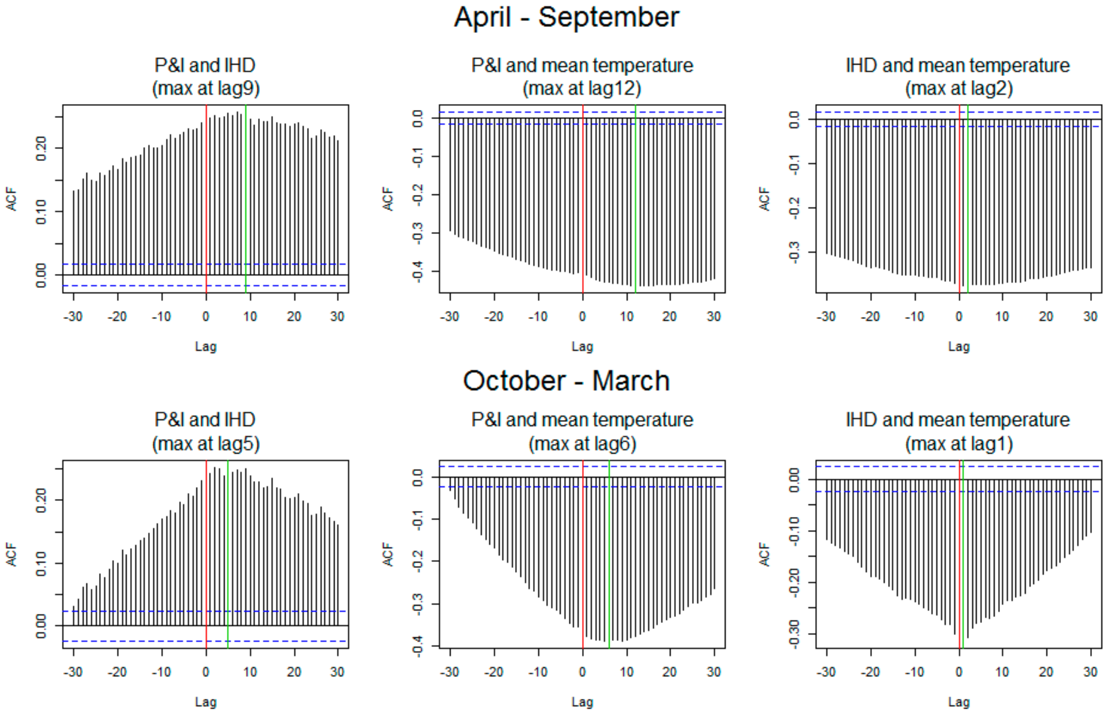


**Figure S4.** Cross-correlations among population aged 15–64.

## 7. Increased Degrees of Freedom for the Splines for Lag and Temperature

Figure S5 show the estimated delayed effects of extreme temperatures on IHD for population aged 65 years or older and age of 15–64. Japan, 1973–2012. As we increased the degrees of freedom from 4 to 8, a secondary peak emerges at approximately day 3 for cold temperatures.

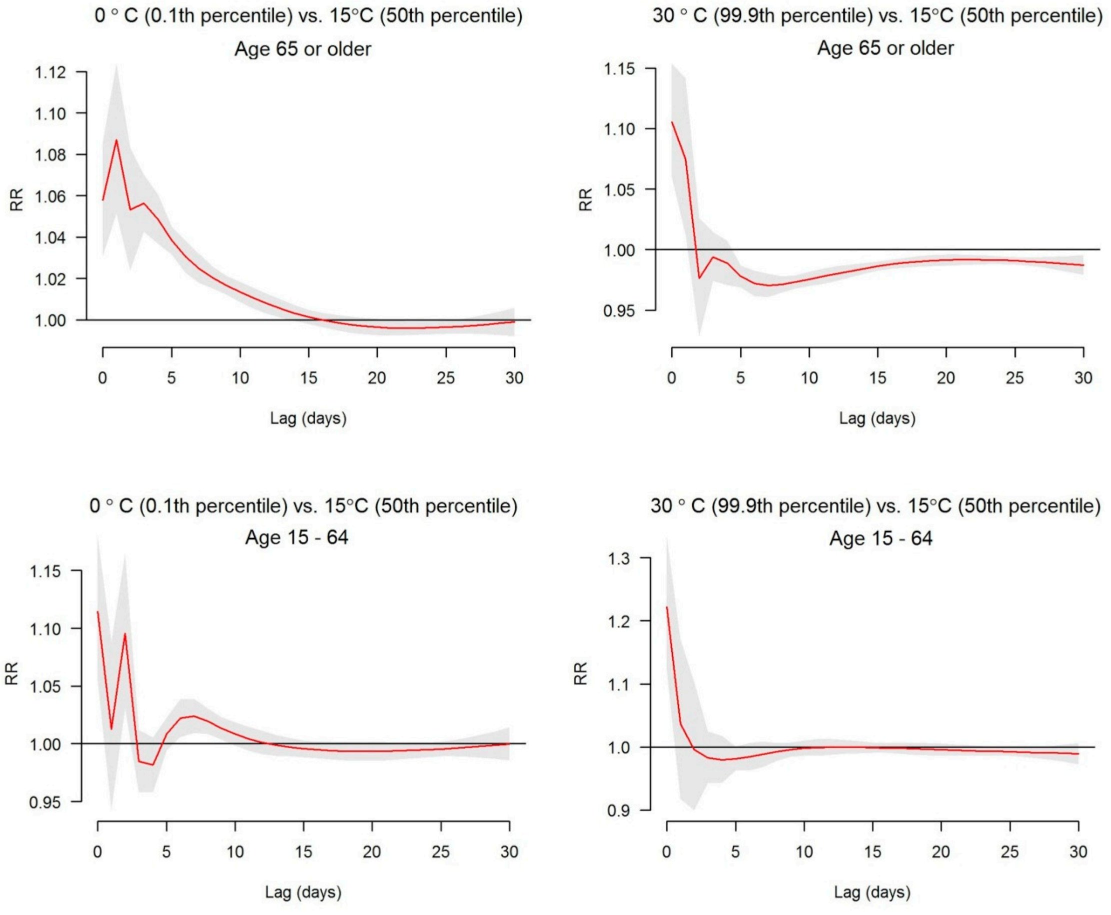


**Figure S5.** Increased degrees of freedom for the splines for lag and temperature.

## 8. Lag Responses of a Cold Effect on IHD from Simulation and Empirical Data

The Figure S4 shows the estimates of cold effect on IHD from simulation and mortality data from Japan. Light green bars are simulated relative risks (RR). The blue lines and grey shades are point estimates and 95% confidence intervals from time series analysis with Japanese mortality data. As aged population accounts for most cases of IHD, time series results for IHD among all age and aged population were very similar with simulated results.

### *8.1. Estimations of Event Times for Simulations*

The initial simulation for the impact of cold on IHD (as described in Figure S1 above) was projected based on the cold season when influenza epidemics occur. In order to simulate the reference values to compute RR, cases during warm season/non-epidemic season were simulated. For the event probability estimates during non-epidemic seasons, Transition 2 and 3 (transitions to influenza infection and IHD) were restated based on case reports in October 2011 in Japan.

#### 8.1.1. Transition 2: Individuals Infected with Influenza

Based on the diagnosis reports in February 2012, the daily rate during epidemics was estimated as 0.0104 (1.04%) ≈ 410,000 ÷ 1,363,000 ÷ 29 days (as stated in Figure S1). The probability during non-epidemic was then estimated as 0.000029 (0.0029%) ≈ 0.0104 × 0.0028 as the national sentinel surveillance reported that influenza-like illness (ILIs) incidence in the preceding October

2011 was 0.28% of February 2012 [7]. Again, since incubation period of influenza is on average 2 days [3], the first 2 days were assumed to be one-fifth of the mean rate.

#### 8.1.2. Transition 3: Individuals to IHD

The daily rate was estimated 0.00049% or 0.049% based on the outpatient survey which was conducted in October 2011, by the Japanese Ministry of Health, Labor and Welfare [4].

### *8.2. Estimates from Time Series Analysis*

Since the cases for non-epidemic season were simulated based on the reports of October 2011, the mean temperature of that time (17.5 °C) was set the reference temperature to cold effect (0 °C) (see Figure S6).

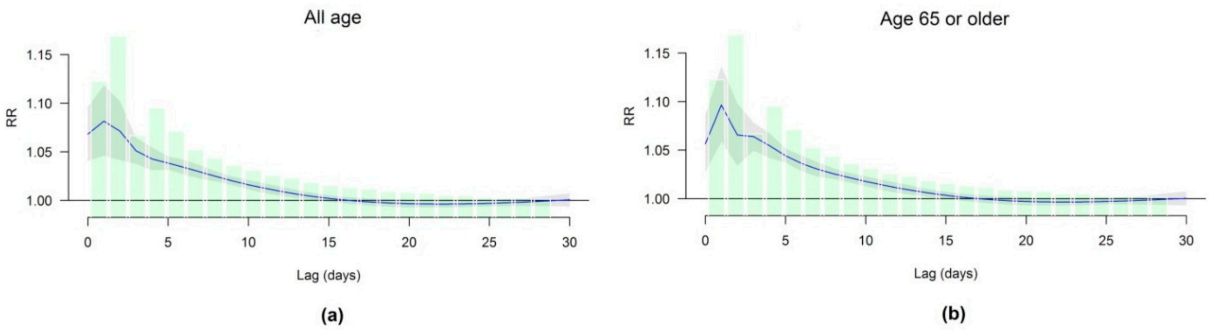


**Figure S6.** Lag responses for estimated risks of a cold effect on IHD based on simulation and empirical data for (**a**) all age group and (**b**) age 65 or older.

## References

1. Fujikawa, A.; Suzue, T.; Jitsunari, F.; Hirao, T. Evaluation of health-related quality of life using EQ-5D in Takamatsu, Japan. *Environ. Health Prev. Med.* **2011**, *16*, 25–35.
2. National Federation of Health Insurance Societies. Report for the Trend of Common Cold, Influenza, and Seasonal Disease 2012. Available online: http://www.kenporen.com/study/toukei_data/pdf/chosa_h24_12.pdf (accessed on 12 December 2014).
3. Centers of Disease Control and Prevention. Clinical Signs and Symptoms of Influenza. Available online: http://www.cdc.gov/flu/professionals/acip/clinical.htm (accessed on 13 November 2014).
4. Japanese Ministiry of Health, Labor and Welfare. Summary of Patient Survey, 2011. Available online: http://www.mhlw.go.jp/toukei/saikin/hw/kanja/11/ (accessed on 12 December 2014).
5. Hong, K.W.; Cheong, H.J.; Choi, W.S.; Lee, J.; Wie, S.H.; Baek, J.H.; Kim, H.Y.; Jeong, H.W.; Kim, W.J. Clinical courses and outcomes of hospitalized adult patients with seasonal influenza in Korea, 2011–2012: Hospital-based influenza morbidity & mortality (HIMM) surveillance. *J. Infect. Chemother.* **2014**, *20*, 9–14.
6. Smeeth, L.; Thomas, S.L.; Hall, A.J.; Hubbard, R.; Farrington, P.; Vallance, P. Risk of myocardial infarction and stroke after acute infection or vaccination. *N. Engl. J. Med.* **2004**, *351*, 2611–2618.
7. National Institute of Infectious Diseases. *Sentinel-Reporting Diseases (Weekly) Number of Cases per Sentinel by Week, Prefecture, and Sex*; Part 27; National Institute of Infectious Diseases: Tokyo, Japan, 2013.

© 2016 by the authors; licensee MDPI, Basel, Switzerland. This article is an open access article distributed under the terms and conditions of the Creative Commons by Attribution (CC-BY) license (http://creativecommons.org/licenses/by/4.0/).
